# A novel classification method of lymph node metastasis in colorectal cancer

**DOI:** 10.1080/21655979.2021.1930333

**Published:** 2021-05-23

**Authors:** Jin Li, Peng Wang, Yang Zhou, Hong Liang, Yang Lu, Kuan Luan

**Affiliations:** a College of Intelligent Systems Science and Engineering, Harbin Engineering University, Harbin, Heilongjiang Province, China; b Department of Radiology, Harbin Medical University Cancer Hospital, Harbin, Heilongjiang Province, China; c College of Information and Electrical Engineering, Heilongjiang Bayi Agricultural University, Daqing, Heilongjiang Province, China

**Keywords:** Colorectal cancer, lymph node, metastasis, deep transfer learning

## Abstract

Colorectal cancer lymph node metastasis, which is highly associated with the patient’s cancer recurrence and survival rate, has been the focus of many therapeutic strategies that are highly associated with the patient’s cancer recurrence and survival rate. The popular methods for classification of lymph node metastasis by neural networks, however, show limitations as the available low-level features are inadequate for classification, and the radiologists are unable to quickly review the images. Identifying lymph node metastasis in colorectal cancer is a key factor in the treatment of patients with colorectal cancer. In the present work, an automatic classification method based on deep transfer learning was proposed. Specifically, the method resolved the problem of repetition of low-level features and combined these features with high-level features into a new feature map for classification; and a merged layer which merges all transmitted features from previous layers into a map of the first full connection layer. With a dataset collected from Harbin Medical University Cancer Hospital, the experiment involved a sample of 3,364 patients. Among these samples, 1,646 were positive, and 1,718 were negative. The experiment results showed the sensitivity, specificity, positive predictive value (PPV) and negative predictive value (NPV) were 0.8732, 0.8746, 0.8746 and 0.8728, respectively, and the accuracy and AUC were 0.8358 and 0.8569, respectively. These demonstrated that our method significantly outperformed the previous classification methods for colorectal cancer lymph node metastasis without increasing the depth and width of the model.

## Graphical abstract

**Figure:**
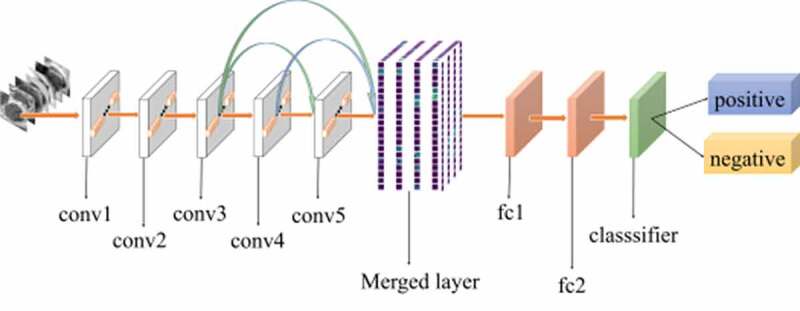

## 1. Introduction

Colorectal cancer (CRC) is the second and third most common cancer diagnosed in men and women, respectively, and it is the second most deadly cancer of all types [1]. The incidence and mortality rates of CRC have been rapidly rising over the last two decades. There was 1,000,000 new cases of CRC and 529,000 CRC-caused deaths in 2002, and the numbers rose to 1,800,000 and 881,000 in 2018 [2–5]. It has become a heavy burden in global health [6]. In the Asia-Pacific region, CRC has clearly developed into a grave health threat [7]. CRC incidence has been rapidly rising among adults under the age of 50 [8], but the outcomes of colorectal cancer treatment have remained unsatisfactory over the last decade [9]. Hence, it is necessary to make appropriate prevention and treatment plans for CRC.

In clinical practice, the presence of CRC lymph node metastasis (LNM) is a significant factor contributing to CRC prevalence [10], and it is important to determine whether LNM is present in patients with CRC because therapeutic strategies differ for patients with and without metastasis [11]. For cases of CRC with LNM, surgical resection accompanied by lymph node (LN) dissection is necessary, whereas endoscopic resection is more appropriate for cases without LNM [11]. Therefore, accurate detection of LNM is critical for the selection of therapeutic plans for CRC patients [12]. In addition, LNM is one of the determinants of the patient survival rate and the primary reason for CRC recurrence [12,13]. CRC patients with LNM have a 5-year survival rate within 50–68%, with a higher risk of loco-regional recurrence. However, for patients without LNM, the 5-year survival rate increases to 95%, and the risk of loco-regional recurrence is relatively lower [14,15]. As a result, the classification of CRC LNM is critical for the preoperative treatment plan. To date, the tumor-node-metastasis (TNM) staging system is the most widely used system in the classification of CRC LNM [16]. The TNM staging system, devised by the American Joint Committee on Cancer (AJCC) [17], is currently the most widely accepted system of staging for CRC. This system comprises three main parameters: T, which reflects the depth of bowel wall infiltration by the primary tumor; N, which indicates the involvement of regional LNM; and M, which refers to distant metastasis spread. There are subsets in T, N and M staging, respectively; T, N and M combined with integer or letter represent the condition of primary tumor, LN metastasis and distant metastasis spread, respectively. N staging is the focus of the system. There are five stages in N staging system, including NX, N0, N1, N2 and N3. The suffix of N represents the extent of lymph node metastasis: NX denotes that the regional lymph nodes cannot be assessed; N0 represents no regional lymph node metastasis, and N1~ N3 represents the increasing involvement of regional lymph nodes. However, the diagnostic efficiency by the TNM staging system remains insufficient [18] and could not support selection of a preoperative treatment plan [19]. Thanks to technological advances, deep learning has been applied to medical image analysis [20,21] for breast cancer [22], lung cancer [23], colorectal cancer [24], and cancer metastasis [25]. Nowadays, deep learning has become a powerful tool in cancer diagnosis. Creating a deep learning algorithm from scratch requires a large amount of data. Detection of CRC LNM by deep learning, therefore, also require many previously-verified images, but these images are hardly attainable because few relevant reports or literature about CRC LNM classification are currently available. Currently, different types of medical images have been used for detection of CRC LNM, including images from endorectal ultrasound (ERUS), computed tomography (CT), and magnetic resonance imaging (MRI), and MRI images are preferred to the other two [26–28]. As radiologists could not rapidly review and classify a large number of images [29], the method of ‘clinical and engineering combination’ is employed. In recent years, radionics has been increasingly used in the evaluation of tumors. An MRI-based radiomics model has been used to distinguish tumors from benign tissues and reflect the histological characteristics of rectal cancer [30,31]. Hence, the present study explores the method of CRC LNM classification using MRI.

Since the methods of transfer learning have been classified by Pan et al. [32], many researchers have been attracted to it. Compared with previous methods [33–38], transfer learning does not require a specific amount of labeled data, and it could automatically extract features from raw data. In recent years, transfer learning by utilizing deep learning has become an attractive and active topic in the field of image analysis [39]. Deep transfer learning directly uses a deep pre-trained model that has been trained by a large-scale dataset (e.g., ImageNet) to transfer the knowledge from the source domain to the target domain.

Deepak et al. presented a method for the classification of brain tumors on MRI images [40]. They modified and fine-tuned a GoogleNet pre-trained model to extract features, and the experiment result showed that classification accuracy was 98%. Cheng et al. proposed a method for the classification of abdominal ultrasound images using deep transfer learning with VGGNet [41]. In their method, the weights of the first 13 convolution layers of the model were frozen, and then used for feature extraction; their experimental results demonstrated that deep transfer learning was more effective for classification of abdominal ultrasound images than other methods. Ragab et al. [42] used the AlexNet pre-trained model for feature extraction, with the support vector machine (SVM) as a classifier. In these above-mentioned methods [40–42], the features of the final classification were extracted by fine-tuning the pre-trained model. The advantage of this extraction method is that features could be extracted and transferred layer by layer. The extracted features are transformed from low-level to high-level, and the high-level features are used for classification. However, this will lead to a lack of feature richness. Although the pre-trained model could extract high-level features, the features are short of diversity and it is likely to lose many useful features. Furthermore, though fine-tuning of the pre-trained model is a simple and effective, numerous extraction attempts are made before suitable parameters are identified. Even though there is currently a grid search method [43], the parameters are related to the result of splitting the original dataset, and the grid search is time-consuming. More parameters indicate more candidate values and hence larger time consumption.

As more classification methods are developed, it turns out that the richness of features is essential to a medical image classification system [44]. In these classification systems, the features extracted from a deep model were combined with the traditional features, then the features were validated on some specific datasets and achieved productive results. The process of feature fusion contains handcrafted features, which may affect the accuracy of the method. Hence, we need a new automated approach to reuse the abandoned features (low-level features) and then combine them with high-level features for final classification. The features extracted by a pre-trained model are divided into low-level and high-level features by nature [45]. The features extracted at the low level have higher resolution, as well as more information on the location and spatial detail, e.g., points, lines, or edges. The features extracted from the high level exhibit greater semantic information. If the low-level features are combined with high-level semantic features, the classification effect can be improved. Similar methods have been used in the field of medical imaging [46,47].

Inspired by these successful studies, we proposed a classification method that combines low-level and high-level features to classify the CRC LNM. This study demonstrated a method for classification of CRC LNM medical images. The method is known as feature multi connection (FMC), which resolves the problem of reusing of low-level features, and combines low-level features with high-level features into a new feature map for the final classification. All features are extracted automatically by the pre-trained model. Automation avoids manual handling that can affect feature classification results. The method had a larger number of transfer features so that it could improve the features to be reused. Besides, in the proposed method, a merged layer was created to merge all transmitted features, and the dimension of the merged layer was determined by five comparative experiments. The major advantage of this method is that it does not require fine-tuning of all parameters of the pre-trained model. Moreover, the interpretability of the method is important in medicine for proper analysis and diagnosis. The convolutional neural network (CNN) is the core of many pre-trained models, but the CNN remains a black box difficult to interpret. Therefore, in the present work, the lesions’ heat-map was utilized to solve the problem of interpretability.

First, the FMC method based on the structure of AlexNet was presented, and real data were used to build a dataset; then, the results of seven automatic classification methods and four radiologists were obtained; through experiments, these results compared with the result achieved by the FMC method from six aspects: sensitivity, specificity, positive predictive value (PPV), negative predictive value (NPV), accuracy, and AUC. The experiment showed that the method proposed in this study significantly outperformed the previous classification methods in CRC LNM classification without increasing the depth and width of the model. In the present work, it was assumed that improving the number of transferred features could increase the performance of the method in CRC LNM classification. Hence, the way of feature transmission was changed based on the AlexNet network structure, and a merged layer was added to combine low-level features with high-level features for final classification so as to achieve a better method for CRC LNM classification.

## 2. Data and methods

### 2.1. Data

**Figure 1.**
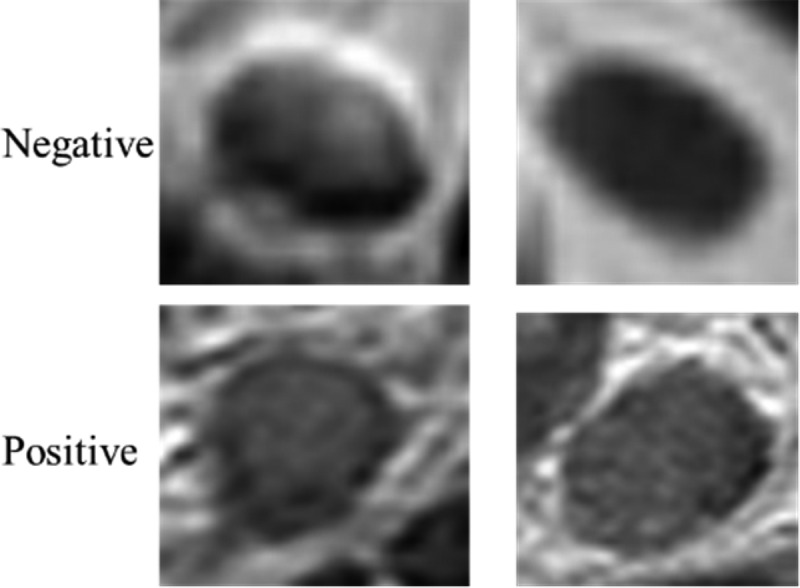
CRC LN. The top row are negative, the bottom row is positive

The data were collected from Harbin Medical University Cancer Hospital between April 2018 and March 2019. The criteria of data were as follows: (I) patients diagnosed with colorectal cancer by endoscopic biopsy and scheduled to undergo surgery within 2 weeks after MRI; (II) patients with no history of treatment before the MRI; (III) patients who had no contraindications and could undergo high-resolution MRI; (IV) patients with at least one mesorectal (peritumoral) or superior mesenteric LNs on MRI; and (V) the standard of all samples was a LN diameter of the lymph node greater than 3 mm. Finally, the dataset contained a sample of 3,364 patients. Among these samples, 1,646 were positive, and 1,718 were negative. All patients underwent 3.0 T magnetic resonance imaging (MRI) scans before surgery using Philips Achieva, with a 16-channel torso array coil. Then, the objective lymph node (LN) in the sagittal, transverse, and coronal images was located. All images used in the present study had been marked as CRC LNs and classified as negative or positive by experienced radiologists. All patches were manually segmented by experienced radiologists, and the image size was based on the lesion to be intercepted. Figure 1 presents the CRC lymph node.

### 2.2. Methods

**Figure 2.**
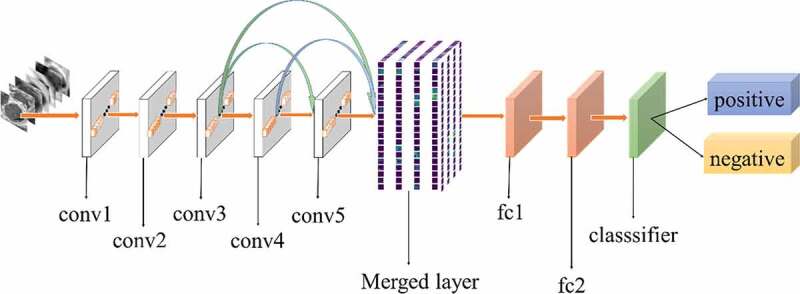
Architecture of our method

Figure 2 shows the architecture of our method. The foundation of our method is AlexNet CNN [48]. AlexNet is the initial landmark breakthrough for image classification. It has significantly outperformed the second runner-up in the ImageNet ILSVRC challenge. Furthermore, AlexNet has displayed strong adaptability to a variety of medical image classification scenarios [49,50]. It has been chosen as the foundation of many medical methods [51–53].

The method proposed in this study was motivated by previous studies [54,55]. The purpose of this method was to increase the flow of features. Traditional deep learning models use the output of the previous layer as the input of the latter layer. This type of connectivity pattern limits the features transfer and results in loss of some useful features. In the present work, a new connectivity pattern was designed and a merged layer was built to merge features of all previous convolutional layers, as shown in Figure 2. Consequently, the formula is as follows:

(1) $$x_{l}=H_{l}\left(x_{n}+x_{n+1}+\ldots +x_{l-1}\right)$$

where *x* is the input, *l* is the location of a layer, *n* is the position of the starting layer, *n < l*, $\left(x_{n}+x_{n+1}+\ldots +x_{l-1}\right)$ is the adding of features from *n* to *l-1.*

Retraining is the process of iterating. The aim of the process is to explore weight *w*, which could minimize the loss of the model:

(2) $$L\left(w,X\right)=\frac{1}{n}\overset{n}{\underset{i=1}{\sum }}l\left(f\left(x_{i},w\right),{\hat{c}}_{i}\right)$$

where X is a training dataset that contains *n* images, $x_{i}$ is the i−th image of X, $f\left(x_{i},w\right)$ is the CNN function that predicts the class $c_{i}$ of $x_{i}$ given w, ${\hat{c}}_{i}$ is the ground-truth class of the i−th image, and $l\left(c_{i},{\hat{c}}_{i}\right)$ is a penalty function for predicting $c_{i}$ instead of ${\hat{c}}_{i}$ . And l was set to the logistic loss function.

The initial weights are from the pre-trained model. In adaptation to CRC LNM medical images dataset, we used SGD to retrain and back-propagation to update weights. The method of updating the weight is as follows:

(3) $$v_{i+1}=0.9*v_{i}-0.0005*\in *w_{i}-\in *\left\langle \;\right.\frac{\partial L}{\partial w}{|}_{w_{i}}{\left.\;\right\rangle }_{D_{i}}$$

(4) $$w_{i+1}=w_{i}+v_{i+1}$$

where v is variable of momentum, and the momentum is 0.95, weight decay is 1e-6, learning rate (LR) is 1e-4. The iteration index is i,∈ is the learning rate, and $\frac{\partial L}{\partial w}{{|}_{w_{i}}}_{D_{i}}$ is the average over the i−th batch Di of the derivative of the objective with respect to w, evaluated at w*_i_* .

During model retraining, local response normalization (LRN) was used to improve generalization. The normalized value $b_{x,y}^{i}$was provided as:

(5) $$b_{x,y}^{i}=\frac{a_{x,y}^{i}}{{\left(k+\alpha \sum_{j=max\left(0,i-n/2\right)}^{min\left(N-1,i+n/2\right)}{\left(a_{x,y}^{j}\right)}^{2}\right)}^{\beta }}$$

where $a_{x,y}^{i}$is the value of i−th kernel output at position $\left(x,y\right)$, *N* is the total number of kernels in the current layer. Other hyper-parameters were set as follows: k = 2, n = 5, alpha = 0.0004, beta = 0.75.

According to [56], features of the first three convolutional layers in a CNN were general. Hence, the proposed method started from the third convolutional layer. The output from the current convolutional layer (the third convolutional layer) was taken as the input of all subsequent convolutional layers until reaching the merged layer. The next convolution layers repeated the operation of the previous ones. All outputs of previous convolutional layers were merged into a feature map, which would be considered as the final input. The merged layer reduced the dimension of the final feature map and extracted the most useful features for classification. Then it was transmitted to the dense layer until the classification layer. The proposed method could improve the performance of classification by reusing features in a better way and avoiding loss of useful features. Table 1 shows the pseudo-code of the merged layer algorithm, where h,w,c represent the height, width, and channels of the input image, respectively; f denotes the features of the convolutional layer and the footnote of f indicates the number of the convolutional layer.

**Table 1.** Pseudo-code of the method algorithm

Algorithm: Method algorithm
1 : Input(h, w, c); 2 : F _output_, F _total,_ f _1,_ f _2 …_ … f _n_ ; 3 : f _1_ (Input(h, w, c)) ; 4 : f _2_ ; (f_1_) ; 5 : f _3_ (f_1_ + f_2_); … … 6 : f _n_ (f _1_ + f _2_ + … … +f _n-1_) ;
7 : F _total_ (f _1_ + f _2_ + … … + f _n_) ; 8 : Δ ‘+’ indicates concatenate operation 9: Δ ‘’ indicates convolution operation
10: F _output_ [∑ (F _total_) ^p^] ^1/p^ ;
11 : return F _output_

To improve the interpretability, the lesions’ heat-map was used to visualize the region of features that were extracted by the method. The heat-map was drawn from the outputs of the final convolutional layer. Formally,

(6) $$M_{c}=\underset{k}{\sum }w_{c,k}f_{k}$$

where $M_{c}$ is the sum of classification feature weights, $f_{k}$ is the kth feature map, $w_{c,k}$ is the weight of the final convolutional layer for feature map k leading to lesions c.

## 3. Experiment

**Figure 3.**
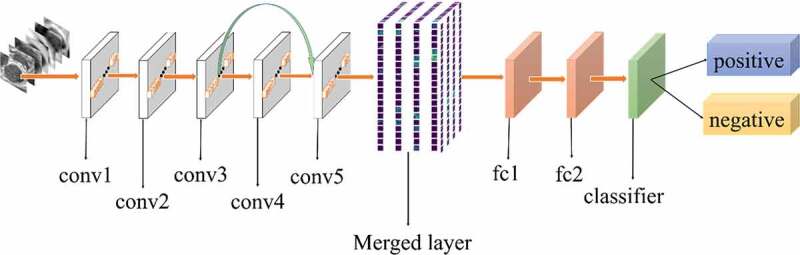
Structure of AlexNet-A

**Figure 4.**
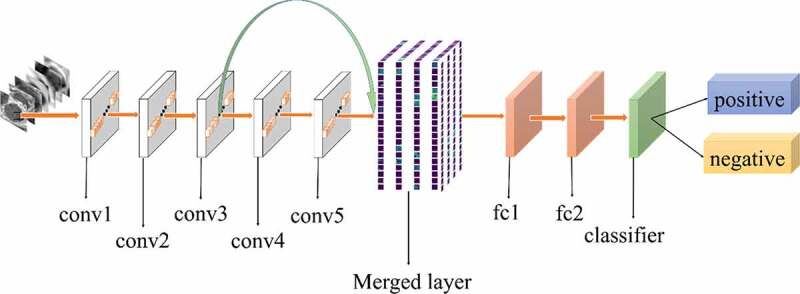
Structure of AlexNet-B

**Figure 5.**
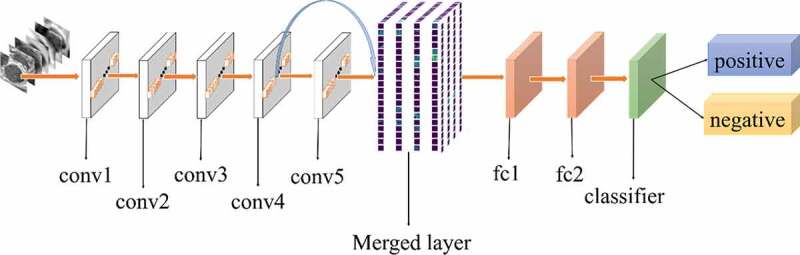
Structure of AlexNet-C

**Figure 6.**
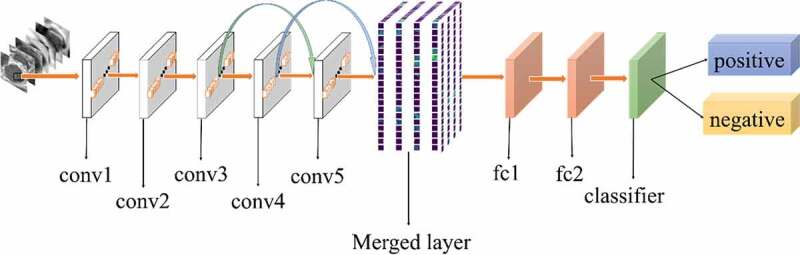
Structure of AlexNet-D

**Figure 7.**
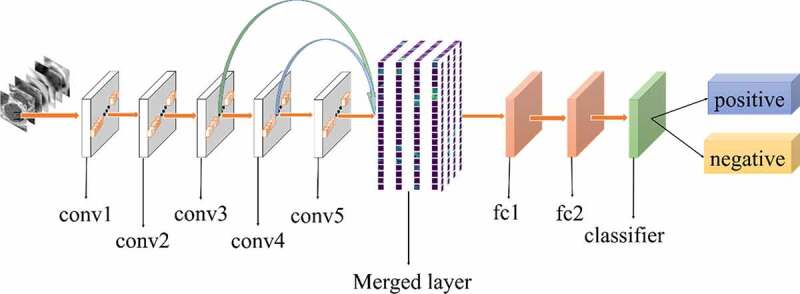
Structure of AlexNet-E

All experiments of the present study used the same dataset. The dataset was randomly spilt into a training set (80%), a validation set (10%) and a test set (10%). The correct width of the merged layer could improve the ability of representation. Therefore, the width of the merged layer was measured. Classification accuracy was used as a determination metric. We used different widths varying from 32 to 4,096 of the merged layer on the CRC LNM dataset, increasing with a power of 2 each time. Then, the dimension with the highest classification accuracy was selected. Then, based on the previous experiment, the effects of different connection patterns on the classification results were tested. There were five styles, as shown in Figures 3–7. In Figures 3 and 4, additional input of the conv5 layer and merged layer were taken from the output of the conv3 layer, named AlexNet-A and AlexNet-B, respectively. In Figure 5, the output of the conv4 layer and the conv5 layer was used as the input of the merged layer, named AlexNet-C. We defined Figure 6 as AlexNet-D. The output of the conv3 layer was seen as an additional input of the conv4 layer, then the output of conv4 and conv5 as the last input to the merged layer. The last structure was AlexNet-E, as shown in Figure 7, the input of the merged layer from the output of conv3, conv4, and conv5, respectively.

To measure the performance of the proposed method for CRC LN metastasis classification, it was compared with the AlexNet model, AlexNet pre-trained model, CNN-AlexNet pre-trained model with SVM, deep domain confusion (DDC) [57], deep adaptation networks (DAN) [58], Resnet152 [59] and Densenet161 [55] for classifying CRC LN metastasis.

In our method, the first step was to load the AlexNet pre-trained model to initialize the weight of all parameters. As previously described [60], the weights represented the extracted features, and according to previous research [56], features extracted by the first three convolutional layers were general features, and their transferability was proved to be better. Hence, the weights of the first three convolutional layers were frozen (fixed), followed by back-propagation to retrain other convolutional layers, as well as fully connected and classifier layers. The method was running on the GPU (NVIDIA, GTX1080Ti). The stochastic gradient descent (SGD) [61] was used as the optimizer. The learning-rate (LR) was set at 1e-4, decay was 1e-6, momentum was 0.95, and the epoch was 200. As CRC LNM classification is a binary classification, the binary_crossentropy was used as the loss function. Other methods for comparison were implemented as specified in literature. In DDC [57] and DAN [58], we selected ImageNet as a source domain and then completed domain adaptation with the target domain (CRC LN dataset).

To explain the internal relationship between input data and the predicted label has been a vital and constant problem in the CNN-based classification models [62]. In the present study, a classification heat-map was employed to improve the interpretability of the model [63]. This experiment contained three steps. First, a model was used to display the last convolutional layer feature-map; second, the feature-map was converted into a heat map.; last, the raw data and heat maps were superimposed into a new image.

In the present work, all methods were implemented by Keras [64], with the backend of TensorFlow [65]. This high-level neural network API written in Python is capable of running on top of TensorFlow, CNTK, or Theano. Furthermore, it can also run seamlessly on the CPU and graphics processing unit (GPU), while TensorFlow is selected as the backend. TensorFlow was created by the Google Brain team for machine learning applications and supports the running the training operation of networks on GPU.

Furthermore, four radiologists with 5-plus years’ clinical experience reviewed the images of CRC LNM, and they independently determined the status of LN. The criteria included irregular borders, heterogeneous signal intensity, and round shape: LN with two or all of them are suspicious. Then, the diagnosis results by the radiologists were compared with our method.

## 4. Results

In this study, a method for CRC LNM classification was proposed. The main idea of this classification method was to improve the number of transferred features, combine low level features with high level features to form a richer new feature map for final classification.

**Figure 8.**
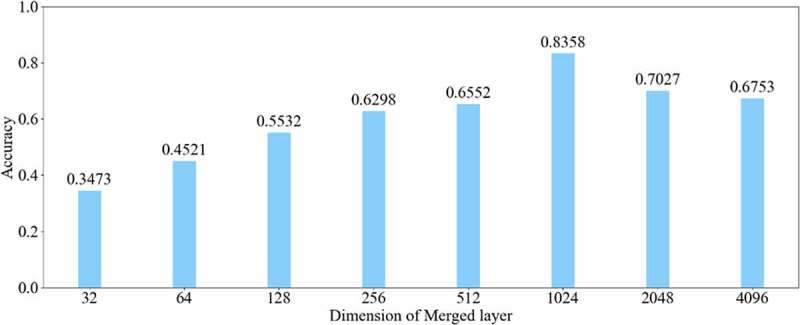
Relationship between accuracy and dimension

The relationship between the accuracy of classification and dimension of the merged layer is shown in Figure 8. The result displayed in 1024 was the most optimal dimension for the merged layer. The results of different connection patterns were shown in Table 2. The classification accuracy from AlexNet-A to AlexNet-E were 0.7598, 0.7725, 0.7968, 0.8088 and 0.8156, respectively.

**Table 2.** Accuracy of five structure

Name	AlexNet-A	AlexNet-B	AlexNet-C	AlexNet-D	AlexNet-E
Accuracy	0.7598	0.7725	0.7968	0.8088	0.8156

**Figure 9.**
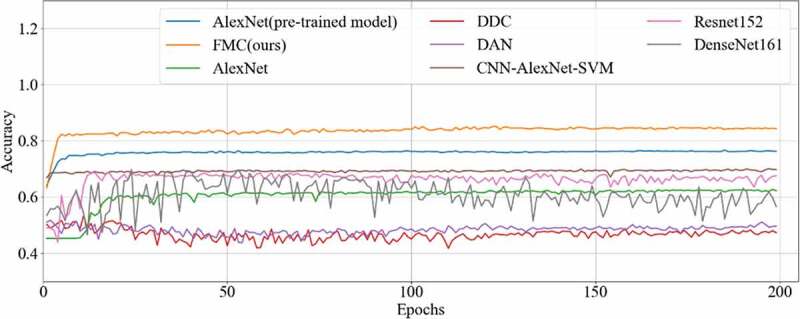
Accuracy curve of six methods on CRC LNM classification

**Figure 10.**
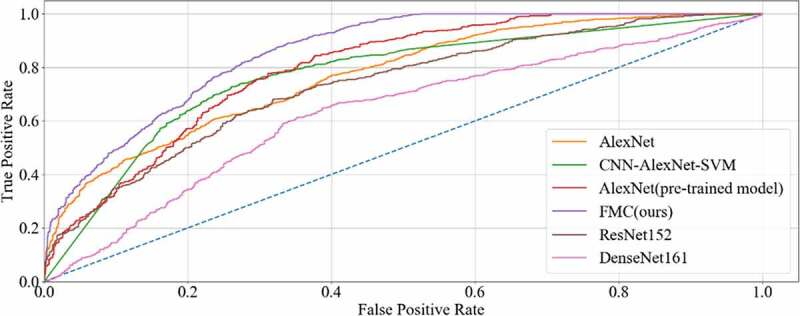
ROC curve of six methods on CRC LNM classification

The final results of all the methods were shown in Table 3, with the accuracy curve shown in Figure 9 and the receiver operating character curve (ROC curve) in Figure 10. The method proposed in this study achieved a classification of 0.8358, and an AUC of 0.8569. The experiment results showed that our method performed better than other methods on CRC LNM classification. Therefore, the MC method could effectively boost the performance of the AlexNet pre-trained model on the CRC LNM classification.

**Table 3.** Classification result on crc lnm metastasis classification

Method	Sensitivity	Specificity	PPV	NPV	Accuracy	AUC
AlexNet model	0.6708	0.6711	0.6714	0.6706	0.6716	0.7696
AlexNet-pretrained model	0.8004	0.7997	0.7992	0.8009	0.7583	0.7941
CNN-AlexNet with SVM	0.7015	0.7015	0.7015	0.7015	0.6920	0.7702
DDC	0.5	0.3478	0.7196	0.1720	0.4670	–
DAN	0.4393	0.4815	0.8444	0.1182	0.4834	–
ResNet152	0.6801	0.6801	0.6801	0.6801	0.6801	0.7327
DenseNet161	0.625	0.625	0.625	0.625	0.625	0.6281
Ours	0.8732	0.8741	0.8746	0.8728	0.8358	0.8569

‘ – ’ indicates the value of AUC less than 0.5

**Figure 11.**
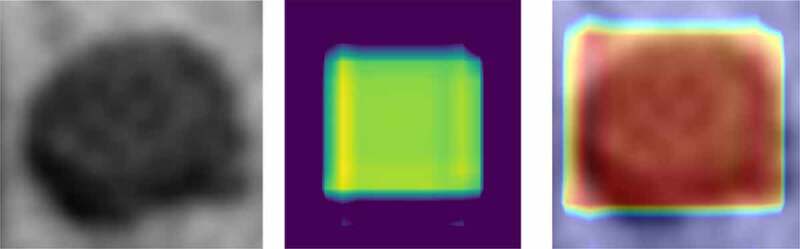
CRC LN classification heat-map. Left is the original image; the middle is the feature heat-map; right is the superimposed image

Although CNN had made an unprecedented breakthrough in a variety of computer imaging techniques, clear interpretation was still needed. Heat-maps improved the interpretability of the CNN model by identifying discriminative regions. As shown in Figure 11, the last convolution layer features a heat-map superimposed on the original MRI image so that the location of the actual lymph node and the region highlighted by the model could be compared. Red regions represent class information, while others correspond to class evidence.

The experimental results diagnosed by four radiologists are shown in Table 4. The sensitivity, specificity, PPV, and NPV from Radiologist 1 were 0.5443, 0.7091, 0.6467, and 0.6142, respectively, while the accuracy and AUC were 0.6279 and 6263, respectively. The sensitivity, specificity, PPV, and NPV from Radiologist 2 were 0.6283, 0.6047, 0.6278, and 0.6616, respectively, while the accuracy and AUC were 0.6432 and 6433, respectively. The sensitivity, specificity, PPV, and NPV from Radiologist 3 were 0.6986, 0.6445, 0.6573, and 0.6613, respectively, while the accuracy and AUC were 0.6717 and 6711, respectively. The sensitivity, specificity, PPV, and NPV from Radiologist 4 were 0.7239, 0.6174, 0.6481, and 0.6896, respectively, while the accuracy and AUC were 0.6694 and 6699, respectively.

**Table 4.** Classification result of four radiologists and our method

Method	Sensitivity	Specificity	PPV	NPV	Accuracy	AUC
Radiologist1	0.5443	0.7091	0.6467	0.6142	0.6279	0.6263
Radiologist2	0.6823	0.6047	0.6278	0.6616	0.6432	0.6433
Radiologist3	0.6986	0.6445	0.6573	0.6613	0.6717	0.6711
Radiologist4	0.7239	0.6174	0.6481	0.6896	0.6694	0.6699
Ours	0.8732	0.8741	0.8746	0.8728	0.8358	0.85697

## 5. Discussion

Based on the results of the experiment, 1024 is the optimal dimension of the merged layer for CRC LNM classification, as shown in Figure 8. Hence, 1024 dimensions were adopted for the merged layer in our method. The merged layer likes regularization, and all features from previous convolutional layers are regularized in this layer and merged with a large feature-map. Then, the features most useful for classification were selected. It was infeasible to select the whole feature-map as the first dense layer input since it would be harmful for classification. The dense layer will connect all parameters for classification, which means that more feature dimensions will result in more computation. Therefore, selecting an appropriate dimension can improve the performance of classification. Figures 3–7 and Table 2 reveal the following findings. First of all, the output of the convolutional layer in the same position was transferred to different positions, which exerted an impact on the classification result, such as AlexNet-A and AlexNet-B. Although there were additional inputs in both structures, the classification accuracy of AlexNet-B was higher than AlexNet-A. Since the output of the conv3 layer had been transferred to the merged layer as the additional input in AlexNet-B, it directly increased the richness of the input for the merged layer. Therefore, it could effectively improve the dimensions of classification features. Moreover, comparing AlexNet-B with AlexNet-C revealed that the additional input of the merged layer from the output of conv4 was more useful than that of the conv3 layer. The features extracted by the convolutional layer at different locations have different effects on the results, with features extracted at the back more specific and useful for CRC LNM classification. This finding was verified by the experimental results of the two structures. In the end, it was also found that the classification accuracy is related to the number of transfer features. As the number of transfer features increased, the classification accuracy improved. In AlexNet-D and AlexNet-E, we increased the number of transfer features and found that the classification accuracy improved. Furthermore, the classification accuracy of AlexNet-E was better than the other four structures.

The method proposed in this study is feasible, considering it is a combination of AlexNet-A to E, and our idea has been validated by CRC LN.

Deep transfer learning could be applied in CRC LNM classification, and the classification result was better than deep learning. Additionally, classification accuracy is related to the number of features on CRC LNM classification. As shown in Table 3 and Figure 9, our method has the highest accuracy among all classification methods. Since the CRC LN data could not be used in AlexNet trained from scratch, the AlexNet model performed worse than others in the classification task. The parameters of AlexNet could not be fitted by the limited CRC LN data. The AlexNet pre-trained model and CNN-AlexNet with SVM utilized different classifiers to classify features that were extracted by pre-trained AlexNet CNN. Therefore, the final classification results were distinct. Our method changed the connection mode and increased the number of feature transfers so that the final feature map contained rich classification information, which was helpful in improving the classification performance.

Domain adaptation was proved useless for CRC LNM classification. In previous years, domain adaptation was the main idea of transfer learning or deep transfer learning [66–68]. The method of domain adaptation involves mapping the source domain (lots of labeled data) and the target domain (little or no label data) to a high dimension space (e.g., reproducing kernel Hilbert space (RKHS) [69]) so that the distribution of data from the two domains is consistent, and then the maximum mean discrepancy (MMD) [69] is used to measure the discrepancy between datasets. However, the method is not easy to understand, and additional calculation was required, posing obstacles to medical technicians. Although Tzeng et al. [57] and Long et al. [58] performed well in Office31 [70], they still could not outperform our method in CRC LNM classification. Classification features are the same in the three datasets of Office31. Therefore, the features extracted from one dataset could be transmitted to the other two. Additionally, domain adaptation could improve the performance of transfer; however, there are some differences between our dataset and ImageNet, such as data distribution, channels, and classification features. Experimental results showed the domain adaptation underperformed on CRC LNM classification. Therefore, the features from the source domain could not be transmitted to our dataset by domain adaptation.

The method proposed in this study is effective in CRC LNM classification and better than other methods, as shown in Table 3. By changing the convolutional layers connection, acquiring more input (features) for the next layer than traditional connection, our method could improve feature reuse and prevent loss of classification information. All features of the previous convolutional layers are transmitted to the merged layer before being merged into the feature map to support classification. In addition to this, the width and depth of deep model architecture did not change, and the parameters of the model did not increase. Therefore, more classification features could be acquired by retraining a small number of parameters, and the deep model could be applied to a small dataset of medical images. Finally, the experimental result confirmed that our method could improve the classification accuracy and is easy to comprehend. Weights of convolutional layers of the general features were frozen, and the rest parameters were retrained so that the final features could be useful for the classification, thereby improving the number of acquired features and merging all features except frozen convolutional layers to improve classification performance.

As shown in Figure 11, the visualization experiment could show the model-focusing region of the input image. The classification heat-map represents evidence of the CNN model-based classification and could assist in clinical decision-making by directly identifying the region of interest.

To the best of the authors’ knowledge, accurate detection of CRC LNM could provide reference indicators for design of treatment strategies and prognosis evaluation. In this study, the experimental data were checked in a node-by-node manner by the radiologists and pathologists to ensure that all data were properly classified, and to guarantee high reliability for data analysis. As shown in Table 4, Radiologist1’s sensitivity, specificity, PPV, NPV, accuracy and AUC were 0.5443, 0.7091, 0.6467, 0.6142, 0.6279 and 0.6263, respectively. Radiologist2’s were 0.6823, 0.6047, 0.6278, 0.6616, 0.6432 and 0.6433, respectively. Radiologist3’s were 0.6986, 0.6445, 0.6573, 0.6613, 0.6717 and 0.6711, respectively. Radiologist4’s were 0.7239, 0.6714, 0.6481, 0.6896, 0.6694 and 0.6699, respectively. The status of LN was a major basis for later treatment, but the results of the current preoperative evaluation were not satisfactory. There were two reasons for this: first, as the histopathological nodes were not matched, the result of imaging nodes was unreliable; second, LN of extremely small sizes could not be distinguished. Therefore, the proposed method could provide an alternative solution to observation by radiologists in identification of CRC LNM.

**Figure 12.**
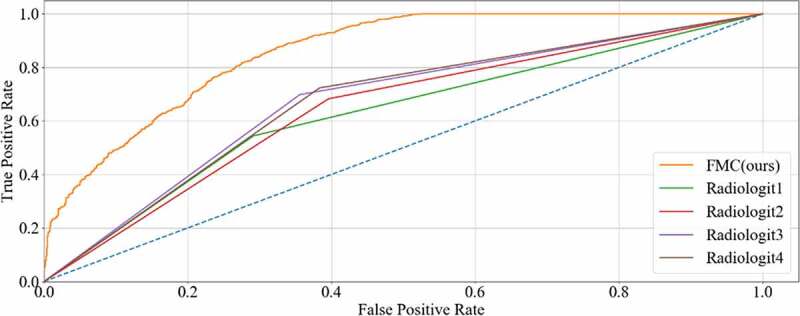
ROC curve of radiologists and our method on CRC LNM classification

Finally, the classification performance of our method was assessed by four radiologists, as shown in Table 4 and Figure 12. The method proposed in this study reached a satisfying outcome. The sensitivity, specificity, PPV, NPV, accuracy, and AUC were 0.8732, 0.8741, 0.8746, 0.8728, 0.8358 and 0.8569, respectively. Compared with the diagnostic accuracy of radiologists (0.6279–0.6717), the accuracy achieved by our proposed method was higher. Hence, the presented method could improve the accuracy of CRC LNM detection and is more credible in the guidance of treatment and prognosis.

Despite the findings, there are limitations in the present work. First, the size of the LNM was limited. In the dataset, in the observation by radiologists, the diameter of the LN must be greater than 3 mm, while LN smaller than 3 mm did not enroll. Second, a benchmark was lacking in this study. Though real data were collected from a cooperative hospital, the data were inadequate to support further research attempts, e.g., training a model from scratch.

## 6. Conclusion

In the present work, a novel feature multi-connection (FMC) architecture was proposed, and the features-merged layer based on AlexNet’s pre-trained model was used to increase CRC LNM classification accuracy. Experimental results showed that this novel method significantly outperformed other existing methods, and improved the accuracy of CRC LNM detection without increasing the depth and width of the model. Therefore, this new method was proved useful for CRC LNM classification and can provide objective second opinions for clinical treatment.

More research efforts will be made in the future to address the current deficiencies of the structure and improve the classification performance of CRC LNM.

## Highlights

1. The number of transferred features was increased.
2. A merged layer was created to form a new feature map;
3. Low-level features were combined with high-level features for classification;
4. Experiment data were real data and preprocessed by radiologists.
